# Targeting Addiction Treatment Engagement: A Canadian Randomized Effectiveness Trial of Remotely Administered Contingency Management for Treatment Attendance: Cibler l’engagement en matière de traitement des dépendances : essai d’efficacité à répartition aléatoire mené au Canada portant sur la gestion des contingences mise en œuvre à distance pour la participation au traitement

**DOI:** 10.1177/07067437261477550

**Published:** 2026-08-27

**Authors:** David C. Hodgins, Jane Yi, Angela Wallace, Laurence Macrae Lynch, Ashley Ethier, Luija Long, Megan E. Cowie

**Affiliations:** 1 Department of Psychology, 2129 University of Calgary, Calgary, Alberta, Canada; 2 Recovery Alberta, 3146 Mental Health and Addiction Services, Edmonton, Alberta, Canada; 3 Department of Mathematics and Statistics, 2129 University of Calgary, Calgary, Alberta, Canada

**Keywords:** contingency management, substance use disorder treatment, effectiveness trial

## Abstract

**Introduction:**

Contingency management (CM) is an evidence-based treatment used as a stand-alone treatment or an adjunct to usual care for individuals with substance use disorders (SUDs). However, its use in clinical practice is limited, particularly in Canada. This study implemented and evaluated the effectiveness of CM for SUDs in 2 outpatient addiction clinics in Alberta.

**Methods:**

A 3-month, 2-armed, parallel-group, unblinded intervention comparing attendance-based CM (*n* *=* 43) with a treatment-as-usual (TAU) control group (*n* = 45). Participants in the CM group were incentivized using a hybrid prize-and-voucher method. Specifically, treatment attendance was rewarded with virtual prize draws, the number of which accrued incrementally based on consecutive attendance.

**Results:**

Participants in CM were retained in treatment for longer durations and attended more treatment sessions with greater consistency compared to participants in TAU. No differences between groups were observed for substance use outcomes or quality of life. The small sample size limits the generalizability of the results.

**Conclusion:**

Our results support the adaptability of CM in the Canadian context to improve treatment retention and attendance. The remote capabilities of CM reduce the barriers to access, allowing for greater reach of this intervention. The results highlight how CM can be tailored to be more economical through its focus on attendance, a focus consistent with improving addiction recovery in Canada.

## Plain Language SummaryTargeting Addiction Treatment Engagement: A Canadian Trial of Remotely Administered Contingency Management for Treatment Attendance

Contingency management (CM) is an intervention that rewards individuals for behaviour changes. Attendance in treatment sessions is important for recovery from Substance Use Disorder. This study found that adding CM to existing outpatient treatment programmes increases the number of sessions a person attended and how long they stayed in treatment.

## Résumé

**Introduction:**

La gestion des contingences (GC) est un traitement fondé sur des données probantes utilisé comme traitement indépendant ou comme traitement d’appoint aux soins habituels chez les personnes atteintes de troubles liés à l’usage de substances psychoactives (TUS). Toutefois, son utilisation dans la pratique clinique est limitée, en particulier au Canada. Cette étude visait à évaluer l’efficacité de la GC pour les TUS dans deux établissements de traitement des dépendances en consultation externe en Alberta.

**Méthodes:**

Une intervention sans insu avec deux groupes parallèles, sur une durée de trois mois, comparait la GC fondée sur la participation (*n* =4 3) à un groupe témoin recevant le traitement habituel (*n* = 45). Les participants du groupe GC étaient encouragés à l’aide d’une méthode hybride de prix et de bons. Plus précisément, la participation au traitement était récompensée par des tirages au sort virtuels, dont le nombre augmentait progressivement en fonction de la participation consécutive.

**Résultats:**

Les participants du groupe GC ont poursuivi le traitement pendant de plus longues périodes et ont participé à plus de séances de traitement avec une plus grande régularité par rapport aux participants dans le groupe du traitement habituel. Aucune différence n’a été observée entre les groupes en ce qui concerne les résultats relatifs à l’usage de substances ou la qualité de vie. La petite taille de l’échantillon limite la possibilité de généralisation des résultats.

**Conclusion:**

Nos résultats appuient l’adaptabilité de la GC dans le contexte canadien pour améliorer la rétention en traitement et la participation à celui-ci. Les capacités de mise en œuvre à distance de la GC réduisent les obstacles à l’accès, ce qui permet d’élargir la portée de cette intervention. Les résultats mettent en évidence la manière dont la GC peut être adaptée pour être plus économique en mettant l’accent sur la participation, en conformité avec le mouvement croissant vers une approche de réduction des méfaits dans le cadre de la réadaptation à la suite de dépendances au Canada.

## Introduction

Timely access to substance use disorder (SUD) treatment continues to be a challenge in most jurisdictions,^1^ complicated by high levels of attrition.^2–4^ Specifically, around 30% to 50% of those who begin treatment leave prematurely.^2,5^ Those not retained in their full treatment course are more likely to relapse^6^ and exhibit greater SU post-treatment.^7,8^ Longer treatment durations are associated with longer periods of abstinence,^9^ reduced likelihood of readmission to outpatient addiction care,^10^ reduced criminal involvement,^7,8^ greater likelihood of full-time employment,^8^ and improved quality of life (QOL).^11^ Overall, there is an urgent need to enhance SUD treatment retention to improve outcomes.

Contingency management (CM) is an evidence-based SUD treatment with a substantial literature base.^12,13^ Although the existing literature is comparatively scant in Canada, current findings from the United States have shown the positive impact of CM on treatment outcomes,^14–17^ including retention.^18,19^ However, despite extensive research CM's uptake in Canada is slow, and it often goes unused in clinical practice.^20,21^ This study implemented and evaluated CM in 2 outpatient addiction treatment programmes in Alberta. It is hoped that CM will be integrated with SU treatment to increase retention, thereby translating into better outcomes.

### Contingency Management

CM is an operant conditioning-based behavioural intervention where patients receive rewards, or incentives, contingent upon meeting a pre-defined specific behavioural goal. Goals are often related to SU, aspects of treatment adherence, or attendance.^13^ A substantial body of literature supports the use of CM in treating various populations with SU concerns.^12,16,22,23^ This includes evidence of the long-term sustainability of effects, with a recent meta-analysis finding a greater likelihood of abstinence 1-year following CM compared to both general outpatient addiction treatment and specific evidence-based treatments (e.g., CBT).^22^ A recent study reported reduced mortality over the same time period among people with stimulant use disorder receiving CM.^24^

CM's efficacy is most often investigated within the context of incentivizing abstinence confirmed through biological testing (e.g., urine samples). Other objectives, like attendance, have also been studied to decrease costs and the logistical challenges of monitoring abstinence,^16,25^ which have been identified as implementation barriers^25–27^ CM providers have also expressed a preference for focusing on harm reduction outcomes versus abstinence.^28,29^ A recent meta-analysis^16^ found that CM incentivizing attendance improved attendance and, to a lesser degree, abstinence.

The provision of CM has also been adapted from in-person to an online or mobile format. Due to the logistics of reliably testing for abstinence (e.g., at-home breathalysers and transdermal patches), most remote CM studies target alcohol^30–32^ and nicotine use,^33–35^ with fewer studies of other drugs.^36,37^ Importantly, this research has demonstrated comparable effects on abstinence between online and in person-delivered CM.^36^ However, more research is needed to assess the efficacy of remotely incentivizing attendance. This is particularly relevant in Canada, where, in recent years, an expanded definition of addiction recovery includes a person-centred approach that does not solely prioritize abstinence-based treatment.^38,39^ Therefore, CM, which incentivizes a non-substance-based goal, like remote attendance, is in line with the current trajectory of addiction treatment focus.

### Current Study

This study evaluated CM effectiveness in 2 outpatient addiction programmes. Our primary outcome was to understand whether CM improved client retention in treatment-as-usual (TAU; H1). Our secondary aim was to understand whether attendance (H2), abstinence, and SU (H3) improved over the 3-month study period and 12-month follow up, compared to TAU. Our tertiary outcome (H4) involved assessing change in QOL (A few preregistered outcomes are not presented in this article due to page limitations. These analyses are contained in M. Cowie's dissertation (Cowie, 2024). A comparison of people who received CM remotely versus in person was planned but not conducted due to the onset of the COVID-19 pandemic. Remote visits were far more frequent compared to in-person visits, which ranged from one to 6 visits throughout the intervention period for all participants. Further, in-person attendance was inconsistent among participants and was limited to the Edmonton site. Our research question was to examine differences in remote and in-person delivery of CM. However, only 6 participants in the CM and 5 in the TAU group ever completed an in-person study visit. Therefore, due to these reasons, the comparison of remote versus in-person CM was not completed.).

## Methods

### Trial Design

This study recruited participants for a 15-month, 2-armed, parallel-group, unblinded RCT comparing CM to a control group. Participants were allocated based on an equal ratio. Given that incentivization occurred for attendance, it was not possible to blind conditions.

### Participants

**Figure 1.**
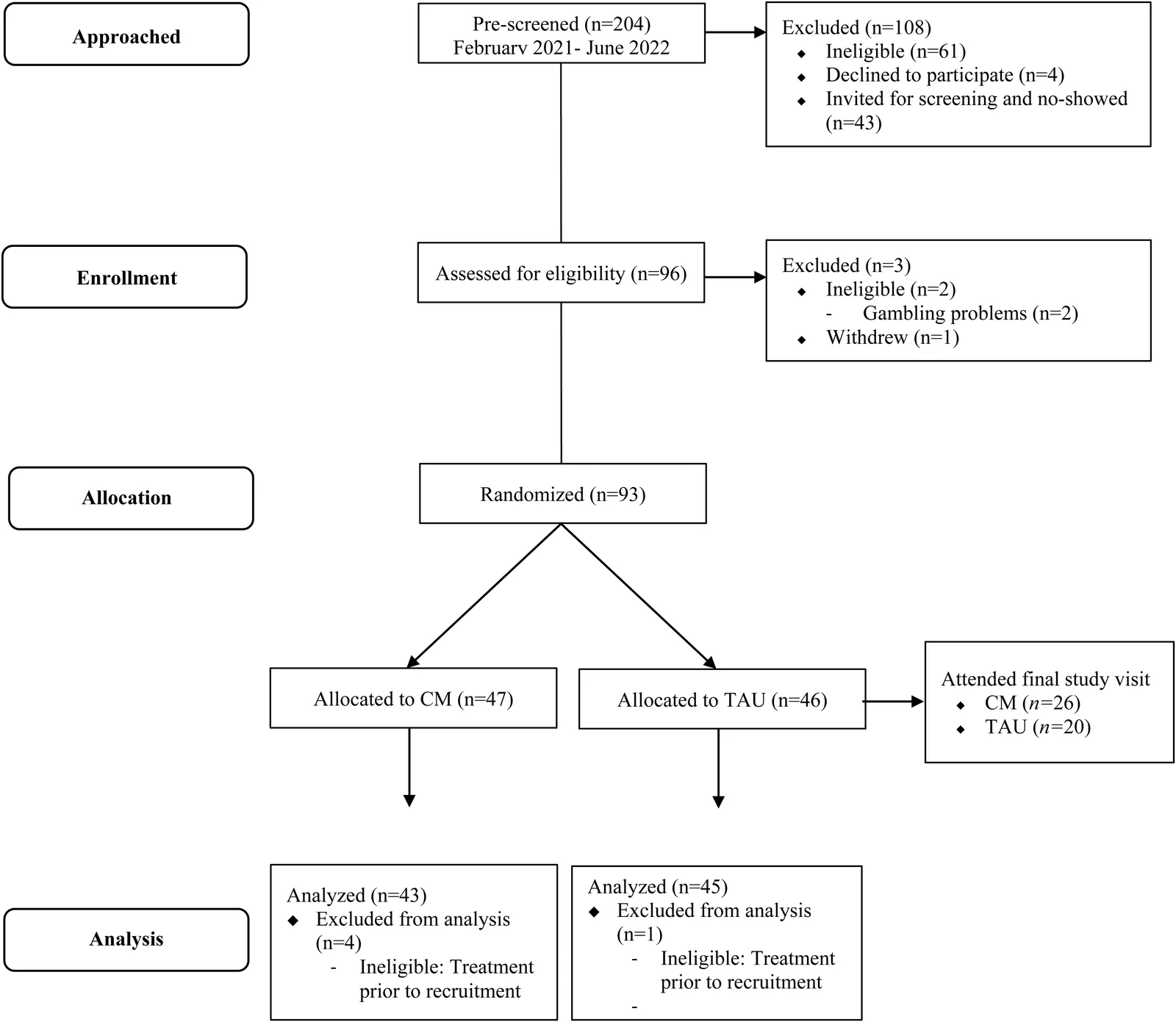
CONSORT flow diagram of the progress of participants in the study.

Participants were recruited from 2 major outpatient addictions clinics in Calgary and Edmonton. In total, 204 individuals were pre-screened for eligibility, and 93 were deemed eligible and consented (see Figure 1). After randomization, 5 participants were discovered to be ineligible because they had already initiated treatment before they were recruited and were subsequently excluded. About 43 participants randomized to CM and 45 from TAU were ultimately included in the analysis. This study was approved by the Research Ethics Board of the University of Calgary in Calgary, Alberta (REB20-0443), and the ClinicalTrials.gov identifier is NCT04544124.

*Inclusion criteria:* (1) About 18 years or older, (2) initiating SU treatment, (3) SU within the past 3 months^40^ (Petry et al., 2005), (4) TAU involving at least weekly attendance, and (5) willing to attend study visits.

*Exclusion criteria*: Due to concerns about the impact of CM prize draws on individuals with gambling problems,^13^ participants with past or current gambling problems were excluded. Participants who reported imminent plans to enter detoxification, inpatient, or residential treatment, or were facing incarceration or home arrest were excluded, as these circumstances would impact ability to participate.

### Measures

Supplemental Table S6 details the study measures. The National Opinion Research Center (NORC) DSM screen for gambling problems (NODS) assessed gambling problems at pre-screening.^41^ Attendance was captured by chart review. The timeline followback questionnaire (TLFB)^42,43^ assessed SU in the 3-months preceding the screening and weekly during the study. Prescription drugs not obtained through a personal prescription to the participant were included. Participants also completed the DSM-5 SUD Checklist (past year) during the screening.^44^ The World Health Organization^45^ QOL-BREF (WHOQOL-BREF) assessed QOL every 2 weeks: physical (α = .78), psychological (*α* = .83), social relationships (*α* = .62), and environment (*α* = .76).

### Outcomes

*Primary outcome*: Retention in TAU was defined as the number of days between the first and last attended treatment session.^40,46^

*Secondary outcomes*: The longest consecutive weeks attended and absent from scheduled treatment, and the total number of treatment sessions attended were extracted from charts.^25,47^ The TLFB provided the number of substance-absent days^47^ for the intervention and follow-up periods. Changes in QOL were measured from baseline to follow up.

### Procedure

*Recruitment and randomization procedures*: Recruitment occurred via referral from clinic counsellors, who conducted initial individualized treatment planning with clients. The randomization sequence used the RStudio “blockrand” package.^48^ Although past literature has demonstrated that CM is effective for individuals whose sex assigned at birth is male or female,^49^ some studies have shown better outcomes for females.^50^ Therefore, participants were randomly allocated using randomly permuted block sizes (2, 4, 6, and 8) stratified by sex (male and female) and site (Calgary and Edmonton).

*Study conditions*: All participants met with study staff weekly, either in-person or through Zoom, to complete study measures on REDCap. All participants were provided with a $30 gift card for attending their final study session during the intervention (week 12).

*Treatment as usual*: All participants received individualized TAU, mostly provided remotely due to COVID-19. The participant and counsellor created an individualized treatment plan, incorporating the participant’s needs and wishes. Treatment plans were highly variable and were typically revised according to participant progress. Treatment was open-ended, but very few treatment plans extended beyond the 12-week intervention phase of the project. TAU could include one-on-one counselling, psychiatry appointments, various group programmes, and attendance at the Rapid Access Addiction Medicine Clinic (Calgary only), as needed. Other than the Day Program, groups operated as “open” such that one could drop in to any classes that were relevant for the client.

*Contingency management*: To provide CM remotely, a prize draw website was developed that was shared with the participant during CM appointments. These sessions were conducted by a project staff member either remotely or in person, separate from treatment sessions. The staff member verified treatment attendance through clinic attendance records. To retain the autonomy and excitement of an in-person prize draw, an animation was shown in response to each slip that the participant “drew” from the virtual bowl (see reference [51] for details). A hybrid prize-and-voucher method was used.^13,40^ Participants who attended their treatment could draw up to 14 slips from a virtual prize-bowl containing 500 slips. The ratio of prize categories and the monetary amount of prize slips were based on past programmes.^13^ Draws escalated based on attendance (see Supplemental Materials) and reset with absences. CM participants received a primer in which attendance at 2 consecutive weeks of treatment was rewarded with a 1-time guaranteed large prize ($20). Participants who drew a slip worth a monetary amount had the option to save it in their study-managed “bank account” until they chose to redeem it in the form of a gift card. The average earnings were $59.17, with the highest earning being $249.00.

#### Statistical Analysis

With Type I error rate of 0.05, Type II error rate of 0.20, a correlation of 0.30 between baseline and end-of-study measures, and using a conservative effect size estimation of *d* = 0.42,^25,52^ 118 participants per group were targeted.^53^ However, we were unable to obtain this and therefore lack the power to detect smaller effects.

We used IBM SPSS Statistics (V29.0). Negative binomial regression analyses and generalized estimating equations (GEE) were used to assess count and continuous outcomes evaluated at a single point in time post-intervention (i.e., number of days retained; consecutive weeks attended). GEE was used to examine outcomes across the baseline and 3-month intervention period; sex, site, and time were covariates. Time was included as a within-subjects variable. Attendance outcomes (i.e., total number of sessions) were recorded across 3 intervention time points: 1, 2, and 3 months. Given these are count data, a negative binomial model with log link was chosen.^54^ Our SU outcomes were assessed across 7 times: baseline, 1, 2, and 3 months in the intervention period and 3-, 6-, and 12-month follow up. SU count variables were analyzed using a negative binomial model with log link, while proportions used a binary logistic model (i.e., proportion of substance absent days). In the distribution of QOL outcomes (WHOQOL-BREF), we noticed violations in normality and positively skewed distributions. Therefore, a gamma log link model was specified.^54,55^ For GEE models, a first-order autoregressive correlation structure was used. Given the small number of planned comparisons in each analysis, pairwise comparisons using least significant differences (LSD) were chosen, with Bonferroni correction.

Outliers assessed using Cook’s distance, standardized residuals, and plots of standardized residuals and predicted values were found in the number of treatment sessions, but the values are plausible based on scheduled treatment. Therefore, sensitivity analyses were performed by recoding to 1 point higher than the next value. No differences emerged, so original values were retained in the main analyses. For remaining analyses, outliers were recoded as 1 point greater than the previous highest value within each period. GEE uses a maximum likelihood approach, which includes participants with complete data. Little's MCAR test was computed, and as missing data were less than 5% and missing completely at random, no data were imputed.

Analyses were first conducted using an intent-to-treat approach, followed by per-protocol analyses. The first per-protocol analysis excluded those who were in controlled environments who could not attend treatment and potentially unable to consume substances. Differences did not emerge. The second per-protocol analysis was based on whether participants in CM attended at least 1 study visit to receive CM. Those who did not receive CM were regrouped into the TAU group. No differences emerged and therefore, solely the intent-to-treat analyses are reported.

## Results

The analytic sample included 49 participants from the Calgary and 39 from the Edmonton site, and 45 participants in TAU and 43 in CM. In total, 46 out of 88 participants (52%) attended the final study visit at 3-months (*n* *=* 20 TAU, *n* *=* 26 CM). Table 1 details demographic characteristics, which did not differ between groups (see Supplemental Tables S7 and S8 comparing sites and study completers [i.e., attended final study session] and noncompleters). Most frequently, participants reported alcohol use disorder (69%), followed by stimulant (41%), cannabis (5%), and opioid use disorder (9%). Noncompleters were younger and more likely to be in common-law relationships than completers.

**Table 1.** Baseline Demographic Characteristics of Participants by Group.

Baseline Characteristics		Full Sample (N = 88)			CM (n = 43)			TAU (n = 45)			Difference at Baseline	
		*n*	%	*M (SD)*	*n*	%	*M (SD)*	*n*	%	*M (SD)*	Test, *df*	*p*
Age^a^		88		36.92 (11.02)	43		37.35 (11.29)	45		36.51 (10.86)	900.50	.576
Sex^b^												
	Female	48	54.55		24	55.81		24	53.33		0.06, 1	.815
	Male	40	45.45		19	44.19		21	46.67			
Gender^c^												
	Female	46	52.57		22	51.16		24	53.33		1.78	.548
	Male	40	45.45		19	44.19		21	46.67			
	Other	2	2.27		2	4.65		0	0.00			
Marital status^c^												
	Single	51	57.95		28	65.12		23	51.11		4.47	.221
	Married	4	4.55		1	2.33		3	6.67			
	Common law	23	26.14		8	18.60		15	33.33			
	Separated/divorced/widowed	9	10.23		6	13.95		3	6.67			
	Missing	1	1.14		0	0.00		1	2.22			
Ethnicity^c^												
	Indigenous	19	21.59		8	18.60		11	24.44		2.65	.688
	Black	2	2.27		2	4.65		0	0.00			
	White	59	67.05		30	69.77		29	64.44			
	Asian	4	4.55		2	4.65		2	4.44			
	Latin American	3	3.41		1	2.33		2	4.44			
	Missing	1	1.14		0	0.00		1	2.22			
Highest level of education^c^												
	High school or lower	38	43.18		16	37.21		22	48.89		1.58	.681
	Trades or college	28	31.82		16	37.21		12	26.67			
	Bachelor's or diploma below	16	18.18		8	18.60		8	17.78			
	Master's or doctorate	6	6.82		3	6.98		3	6.67			
Employment status^c^												
	Employed	36	40.91		15	34.88		21	46.67		2.24	.532
	Unemployed	31	35.23		18	41.86		13	28.89			
	Student	7	7.95		4	9.30		3	6.67			
	Retired/disabled/leave	14	15.91		6	13.95		8	17.78			
Household income before tax^c^											
	$0–$9,999	6	6.82		1	2.33		5	11.11		6.34	.516
	$10,000–$19,999	9	10.23		4	9.30		5	11.11			
	$20,000–$39,999	22	25.00		13	30.23		9	20.00			
	$40,000–$59,999	11	12.50		6	13.95		5	11.11			
	$60,000–$79,000	9	10.23		3	6.98		6	13.33			
	$80,000–$99,000	4	4.55		3	6.98		1	2.22			
	$100,000+	13	14.77		5	11.63		8	17.78			
	Unsure	14	15.91		8	18.60		6	13.33			
Substance use												
Alcohol use disorder		61	69.13		30	69.80		31	68.90		0.01	.929
Cannabis use disorder		13	14.80		8	18.60		5	11.10		0.98	.322
	Opioid use disorder	8	9.10		3	7.00		5	11.10		0.46	.500
	Stimulant use disorder	36	40.90		20	46.50		16	35.60		1.09	.296
	Other SUD	4	4.54		2	4.70		2	4.40		0.01	.929
	Longest consecutive days abstinent^d^	88		15.55 (18.65)	43		5.74 (0.14)	45		4.65 (0.11)	0.907, 1	.341
	Longest consecutive days of substance use^d^	88		42.00 (34.07)	43		15.35 (0.36)	45		12.71 (0.29)	0.76, 1	.382
	Mean standard drinks^d^	88		9.69 (7.75)	43		9.93 (0.24)	45		9.47 (0.22)	0.05, 1	.831
	Total number of standard drinks^d^	88		366.82 (391.99)	43		134.89 (3.14)	45		110.21 (2.45)	0.90, 1	.344
	Largest number of standard drinks^d^	88		13.10 (9.71)	43		13.86 (0.33)	45		12.38 (0.29)	0.26, 1	.609
	Proportion of days abstinent^a^	88		0.36 (0.32)	43		0.33 (0.33)	45		0.38 (0.32)	881.00	.468
WHOQOL-BREF												
	Environment^a^	88		67.12 (16.11)	43		67.23 (14.30)	45		67.01 (17.82)	937.00	.798
	Social^a^	88		53.60 (22.31)	43		50.19 (23.32)	45		56.85 (21.04)	1.41, 86	.163
	Physical health^e^	88		59.38 (18.63)	43		60.96 (15.46)	45		57.86 (21.29)	−0.79, 80	.217
	Psychological^e^	88		47.30 (20.02)	43		50.78 (16.55)	45		43.98 (22.54)	−1.62, 81	.110

*Note.*^a^Mann–Whitney U test; ^b^Chi square test; ^c^Fisher's exact test; ^d^negative binomial regression; ^e^independent samples t-test; CM = contingency management, TAU = treatment-as-usual.

### Hypothesis 1: Retention in Treatment

*Days retained in treatment*: A negative binomial regression examined treatment retention postintervention while GEE assessed retention rates over time. In total, 85 participants were included (*n* = 44 TAU, *n* *=* 41 CM). Overall, the model indicated a statistically significant effect of group (*Wald*(1) = 10.95, *p* < .001) but not site (*Wald*(1) = 2.78, *p* = .096) or sex (*Wald*(1) = 0.70, *p* = .404). Parameter estimates indicated that those in CM were 2.10 times more likely to have longer treatment retention compared to participants in TAU (B = 0.74, 95% CI [0.30, 1.18], *Wald*(1) = 10.95, *p* < .001). Estimated marginal means (EMM) indicated those in CM were retained in treatment for 62.21 days (*SE* = 10.09) compared to 29.59 days (*SE* = 4.54) in TAU.

In terms of GEE results over time, an effect of group (*Wald*(1) = 15.67, *p* < .001) and time (*Wald*(2) = 17.97, *p* < .001) were observed. There were no effects of site (*Wald*(1) = 0.33, *p* = .566), sex (*Wald*(1) = 1.37, *p* = .242), or group*time interaction (*Wald*(1) = 1.24, *p* = .539). Parameter estimates showed that those in CM were retained nearly 2 times longer compared to TAU (see Table 2). Further, retention was higher during the first month of treatment compared to the second and third months. Despite a non-significant interaction, planned pairwise comparisons between groups revealed that compared to TAU, CM participants were retained for a longer number of days at 1 (*p* < .001), 2 (*p* < .001), and 3 months (*p* *=* .003) (Bonferroni corrected α = 0.017).

**Table 2.** GEE for Retention Across Time.

Effect	IRR	Estimate	*SE*	*df*	Wald Chi-Square	*p*	95% CI	
							LL	UL
Retention								
Intercept	10.20	2.32	0.22	1	108.30	<.001	1.89	2.76
Group: CM	1.84	0.61	0.17	1	12.47	<.001	0.27	0.95
Site: Edmonton	1.12	0.12	0.20	1	0.33	.566	−0.28	0.51
Sex: Female	0.79	−0.23	0.20	1	1.37	.242	−0.63	0.16
Time: 2 months	0.62	−0.48	0.23	1	4.56	.033	−0.93	−0.04
Time: 3 months	0.46	−0.77	0.27	1	8.12	.004	−1.31	−0.24
Group*Time: 2 months	1.23	0.21	0.25	1	0.67	.413	−0.29	0.70
Group*Time: 3 months	1.37	0.32	0.32	1	0.95	.330	−0.32	0.95

*Note.* Reference categories: CI = 95% confidence interval; CM = contingency management; *df* = degrees of freedom; GEE = generalized estimating equation; IRR = incidence rate ratio; LL = lower limit; SE = standard error; TAU = treatment-as-usual, Calgary, male, 1 month; UL = upper limit. About 85 participants were analyzed at month 1 (*n* *=* 44 TAU, *n* *=* 41 CM), 81 at month 2 (*n* *=* 43 TAU, *n* *=* 38 CM), and 77 at month 3 (*n* *=* 39 TAU, *n* *=* 38 CM).

### Hypothesis 2: Treatment Attendance

*Total treatment sessions*: Effects of group (*Wald*(1) = 22.96, *p* < .001) and time (*Wald*(2) = 26.09, *p* < .001) were observed, with no effect of site (*Wald*(1) = 0.10, *p* = .753), sex (*Wald*(1) = 0.03, *p* = .867), or group*time interaction (*Wald*(2) = 0.81, *p* = .667). As hypothesized, CM participants attended 2.34 times as many treatment sessions compared TAU (see Table 3 for GEE results and Table 4 for EMM). Overall, participants attended more treatment sessions during the first compared to the third month of treatment. Despite a nonsignificant interaction, planned pairwise comparisons revealed CM participants attended more treatment sessions at 1 (*p* < .001), 2 (*p* < .001), and 3 months (*p* *=* .002) during the intervention (α = 0.017).

**Table 3.** GEE for Attendance Outcomes.

Effect		IRR	Estimate	*SE*	*df*	Wald Chi-Square	*p*	95% CI	
								LL	UL
Total sessions									
Intercept	2.24	0.80	0.22	1	13.21	<0.001	.37	1.23	
Group: CM	2.34	0.85	0.16	1	29.82	<0.001	.55	1.16	
Site: Edmonton	0.94	−0.06	0.20	1	0.10	0.753	−.46	0.33	
Sex: Female	1.03	0.03	0.19	1	0.03	0.867	−.35	0.41	
Time: 2 months	0.67	−0.40	0.21	1	3.64	0.056	−.82	0.01	
Time: 3 months	0.41	−0.89	0.26	1	12.20	<0.001	−1.39	−0.39	
Group*Time: 2 months	1.18	0.16	0.24	1	0.45	0.501	−.31	0.63	
Group*Time: 3 months	1.27	0.24	0.31	1	0.60	0.441	−.37	0.85	
Retention (longest consecutive weeks attended)									
Intercept		2.20	0.79	0.27	1	8.56	.003	1.30	3.73
Group: CM		2.15	0.77	0.26	1	8.97	.003	1.30	3.56
Site: Edmonton		1.01	0.01	0.26	1	<0.01	.965	0.61	1.69
Sex: Female		1.02	0.02	0.26	1	<0.01	.955	0.61	1.70

*Note.* Reference categories: CI = 95% confidence interval; CM = contingency management; *df* = degrees of freedom; GEE = generalized estimating equation, IRR = incidence rate ratio; LL = lower limit; SE = standard error; TAU = treatment-as-usual, Calgary, male, 1 month; UL = upper limit. About 85 participants were analyzed (*n* *=* 44 TAU, *n* *=* 41 CM).

**Table 4.** GEE for Proportion of Abstinent Days Across Time for Intervention and Follow-Up Periods.

Effect	OR	Estimate	*SE*	*df*	Wald Chi-Square	*p*	95% CI	
							LL	UL
Intervention period								
Intercept	0.97	−0.03	0.29	1	0.01	.921	−0.60	0.54
Group: CM	0.80	−0.23	0.31	1	0.55	.459	−0.83	0.37
Site: Edmonton	0.56	−0.58	0.33	1	3.12	.077	−1.22	0.06
Sex: Female	0.70	−0.35	0.31	1	1.26	.262	−0.96	0.26
Time: 1 month	1.95	0.67	0.21	1	10.81	.001	0.27	1.06
Time: 2 months	2.18	0.78	0.26	1	9.06	.003	0.27	1.28
Time: 3 months	2.51	0.92	0.28	1	10.76	.001	0.37	1.47
Group*Time: 1 month	1.61	0.48	0.29	1	2.74	.098	−0.09	1.04
Group*Time: 2 months	1.67	0.51	0.34	1	2.24	.134	−0.16	1.18
Group*Time: 3 months	1.63	0.49	0.38	1	1.69	.193	−0.25	1.23
Follow-up period								
Intercept	4.12	1.42	0.68	1	4.28	.039	1.08	15.76
Group: CM	1.58	0.46	0.55	1	0.68	.408	0.54	4.64
Site: Edmonton	0.48	−0.73	0.58	1	1.61	.205	0.15	1.49
Sex: Female	0.44	−0.83	0.61	1	1.87	.171	0.13	1.43
Time: 6 months	1.08	0.08	0.15	1	0.29	.593	0.81	1.45
Time: 12 months	1.25	0.22	0.31	1	0.52	.472	0.68	2.29
Group*Time: month 6	0.62	−0.48	0.48	1	1	.319	0.24	1.58
Group*Time: month 12	0.64	−0.44	0.55	1	0.64	.422	0.22	1.89

*Note.* Reference categories: CI = 95% confidence interval; CM = contingency management; *df* = degrees of freedom; GEE = generalized estimating equation; LL = lower limit; OR = odds ratio; SE = standard error; TAU = treatment-as-usual, Calgary, male, baseline; UL = upper limit.

*Longest consecutive weeks attended*: An effect of group (*Wald*(1) = 8.97, *p* = .003) was observed. There was no effect of site (*Wald*(1) = 0.002, *p* = .965) or sex (*Wald*(1) = 0.003, *p* = .955). Participants in CM attended a mean of 4.80 consecutive weeks (SE = 0.85) versus 2.23 (SE = 0.41) for TAU (see Table 3 for GEE results).

### Hypothesis 3: Substance Use

In our GEE analysis for SU, 88 participants were analyzed at baseline (*n* *=* 45 TAU, *n* *=* 43 CM), 76 at month 1 (*n* *=* 37 TAU, *n* *=* 39 CM), 62 at month 2 (*n* *=* 27 TAU, *n* *=* 35 CM), and 56 at month 3 (*n* *=* 24 TAU, *n* *=* 32 CM). For the postintervention period, 41 were analyzed at 3 months, (*n* *=* *17* TAU, *n* *=* 24 CM), 33 at 6 months (*n* *=* *12* TAU, *n* *=* *22* CM), and 21 at 12 months (*n* *=* *6* TAU, *n* *=* *15* CM).

*Proportion of substance absent days during intervention period*: The test of model effects indicated an effect of time (*Wald*(3) = 44.05, *p* *<* .001) but not group (*Wald*(1) = 0.19, *p* *=* .666), site (*Wald*(1) = 3.12, *p* *=* .077), sex (*Wald*(1) = 1.26, *p* *=* .262), or group*time (*Wald*(3) = 2.85, *p* = .416). Parameter estimates indicated the odds of abstinent days increased by 1.95-, 2.18-, and 2.51-times between baseline and months 1, 2, and 3 (Table 4). No other effects were significant, and no planned pairwise comparisons were significant at baseline, (*p* *=* .456), month 1 (*p* *=* .502), 2 (*p* *=* .473), or 3 (*p* *=* .523; α = 0.013; Table 5 for EMM).

**Table 5.** Estimated Marginal Means of Proportion of Days of Abstinence and WHOQOL-BREF QOL by Group and Time for Intervention and Follow-Up Periods.

	Intervention Group			
	CM		TAU	
Outcome Period	*M*	*SE*	*M*	*SE*
Intervention				
Baseline	0.33	0.05	0.38	0.05
Month 1	0.60	0.07	0.54	0.07
Month 2	0.64	0.07	0.57	0.07
Month 3	0.67	0.07	0.61	0.07
Follow up				
3 months	0.75	0.07	0.65	0.08
6 months	0.67	0.10	0.67	0.08
12 months	0.70	0.09	0.70	0.10
Quality of Life				
Physical health				
Baseline	61.20	2.42	57.66	3.03
Week 2	63.69	2.59	58.56	3.26
Week 4	64.43	2.32	64.37	4.55
Week 6	67.16	2.80	61.47	3.47
Week 8	66.73	2.62	61.21	3.76
Week 10	69.04	2.57	59.21	3.35
Week 12	70.30	2.47	62.37	3.93
Period 1	71.27	3.47	61.35	4.78
Period 2	69.90	2.82	64.91	4.17
Period 3	71.22	4.14	65.63	3.37
Psychological				
Baseline	51.10	2.59	43.74	3.33
Week 2	54.48	2.53	50.68	3.84
Week 4	56.85	2.63	53.78	3.73
Week 6	58.61	3.01	56.26	3.67
Week 8	56.02	2.89	54.62	3.73
Week 10	57.02	2.66	49.79	5.21
Week 12	57.82	2.95	55.48	4.45
Period 1	62.17	3.51	51.45	5.03
Period 2	61.67	3.19	52.54	4.96
Period 3	58.53	5.23	53.20	6.30
Social				
Baseline	50.55	3.47	57.08	3.06
Week 2	53.34	3.59	62.19	3.99
Week 4	56.06	3.41	61.33	3.68
Week 6	61.63	3.48	62.75	3.36
Week 8	57.21	3.38	64.79	4.08
Week 10	57.53	3.82	59.00	4.59
Week 12	58.09	3.69	61.73	4.93
Period 1	59.14	4.11	58.73	4.82
Period 2	58.05	4.40	57.15	6.66
Period 3	55.97	5.04	57.32	4.79
Environment				
Baseline	67.64	2.20	67.24	2.59
Week 2	67.41	2.29	67.98	2.54
Week 4	68.27	2.82	69.78	3.23
Week 6	71.23	2.73	67.42	3.04
Week 8	66.41	2.60	68.32	3.04
Week 10	66.60	2.31	67.03	3.14
Week 12	67.35	2.32	68.25	3.17
Period 1	66.17	2.93	62.84	4.39
Period 2	66.72	3.25	68.03	3.82
Period 3	68.07	4.10	62.88	3.41

*Note.* CM = contingency management; TAU = treatment-as-usual; WHOQOL-BREF = World Health Organization (WHO) quality of life-BREF. About 88 participants were analyzed at baseline (*n* *=* 45 TAU, *n* *=* 43 CM), 60 participants were analyzed at week 2 (*n* *=* 27 TAU, *n* *=* 33 CM), 47 at week 4 (*n* *=* 19 TAU, *n* *=* 28 CM), 46 at week 6 (*n* *=* 22 TAU, *n* *=* 24 CM), 43 at week 8 (*n* *=* 16 TAU, *n* *=* 27 CM), 38 at week 10 (*n* *=* 17 TAU, *n* *=* 21 CM), 46 at week 12 (*n* *=* 20 TAU, *n* *=* 26 CM), 34 at follow-up period 1 (*n* *=* 14 TAU, *n* *=* 20 CM), 30 at follow-up period 2 (*n* *=* 10 TAU, *n* *=* 20 CM), and 23 at follow-up period 3 (*n* *=* 7 TAU, *n* *=* 16 CM).

*Proportion of substance-absent days during follow-up*: No effect of group (Wald(1) = 0.080, *p* = .777), site (Wald(1) = 1.607, *p* = .205), sex (Wald(1) = 1.875, *p* = .171), time (Wald(2) = .902, *p* = .637) or a group*time interaction (Wald(2) = 1.016, *p* = .602) were found. Planned pairwise comparisons between group*time revealed no significant differences at period 1 (*p* = .406), 2 (*p* = .973), or 3 (*p* = .984; Tables 4 and 5).

### Hypothesis 4: Quality of Life

Supplemental Table S9 presents the WHOQOL-BREF GEE results. Table 5 includes EMM (see Supplemental Figure S1 for graphs). A significant time effect was found for physical and psychological domains, indicating improvements from baseline at some time periods. No group differences were found when correcting for multiple comparisons (α = 0.005).

## Discussion

Overall, we found that individuals attending outpatient addiction treatment with CM as an adjunct to usual care stayed in treatment longer and attended more sessions compared to those whose usual care does not involve CM. Given that abstinence-based CM can be relatively expensive as it requires collection of biological samples, the results from our study suggest that targeting attendance may offer an efficient solution to improving retention and ensuring consistent treatment attendance, supporting findings from previous research.^25^ Several studies report that CM providers find that incentivizing attendance versus abstinence is also more aligned with treatment when patients have treatment goals for SU and other life areas beyond cessation of SU.^27–29^ Our study also provides evidence for the adaptability of attendance-based CM for remote delivery, which can reduce barriers to treatment access.

The average CM earnings ($59) were unintentionally much lower than recommended in the literature (e.g., $164).^13^ The magnitude of incentives has been shown to be an important factor in promoting abstinence and attendance.^56^ However, despite our low average earnings, CM was still found to be effective. A possible explanation for our low earnings is the frequency of TAU in our participating clinics. Many treatment plans involved once weekly treatment and were less than 12 weeks. Therefore, the escalation of prize draws was perhaps slower and, for some, ended earlier than recommended, yielding less money overall. Future implementations in clinics with similar TAU patterns may require modified prize ratios.

In contrast to our hypotheses and previous literature,^16,25^ we did not find that incentivizing attendance led to a larger reduction in SU. Participants in both conditions showed reductions. We used the TLFB as opposed to biological measures due to the COVID-19 imposed constraints on in-person visits. It is possible that the use of unverified self-reports may have resulted in reporting bias. Importantly, CM studies that have found a relationship between incentivizing attendance and a reduction in SU have found small effect sizes.^16,25^ Therefore, it is possible that our small sample size and high rates of attrition prevented us from detecting an effect. Upon visually examining the graphs of our EMM, the trends consistently favour the CM group for select SU outcomes. Future CM research should recruit larger sample sizes to provide adequate statistical power to detect meaningful effects of both retention and SU. Although CM has been tested in a variety of different drug use types, in-study comparisons of different subpopulations are important. In this study, the original intent was to recruit a larger sample size and to focus on methamphetamine use, which is particularly strongly linked to treatment dropout. However, recruitment of methamphetamine using patients was challenging and precluded our planned subgroup analyses.

Our study also found no significant differences in QOL between CM and TAU, which is consistent with some literature^57^ and in contrast to others.^58,59^ Again, our power to detect effects was low. It is also possible that aspects of the WHOQOL-BREF may have played a role. First, the internal consistency of the subscales in our sample ranged from marginal to good, with only the psychological domain reaching a good level. It is probable that lower levels of reliability further reduced power. Second, the domains of the WHOQOL-BREF may not have been optimal to capture QOL changes. For example, treatment is not likely to rapidly impact individuals’ felt sense of safety in their environment. Therefore, future research may wish to incorporate QOL indices likely to be impacted.

### Strengths and Limitations

The modification from in-person to virtual highlights the adaptability of CM. Moreover, offering gift cards as opposed to prizes from a gift cabinet, as originally planned, reduced the staff resources required to provide incentives.

Regarding limitations, our sample size was small, and our retention rates were low. This represents a significant threat to the generalizability of the study findings. Although we randomized 88 people, only 46 attended their final study visit. It is possible that Zoom fatigue contributed to low retention rates. Although remote care has benefits,^60^ research has also shown that a higher frequency of virtual meetings is related to greater exhaustion.^61^ Hence, participants may not have wanted to attend our virtual study visits following virtual therapy. Some participants also indicated that finding a private space for virtual study visits was challenging.

Further, several aspects of this study were altered in accordance with COVID-19 safety guidelines. This includes the delivery of TAU, which, although altered dramatically, may align with the growth of telehealth and virtual treatment. An additional limitation related to COVID-19 was the removal from the project design of a comparison group incentivizing abstinence tested through biological samples, intended to provide a unique direct comparison of the efficacy of abstinence versus attendance incentives.

## Conclusions

Notwithstanding limitations, this study presents needed information regarding CM's effectiveness in Canada, specifically regarding incentivizing attendance. Further, it provides evidence on the adaptability of CM and suggests that online-delivered CM incentivizing attendance, versus abstinence, is an effective alternative for this population. It is hoped that the promising outcomes from this study will encourage the further investigation of CM as an adjunct to SU treatment in Canada.^20^

## Supplemental Material

sj-docx-1-cpa-10.1177_07067437261477550 - Supplemental material for Targeting Addiction Treatment Engagement: A Canadian Randomized Effectiveness Trial of Remotely Administered Contingency Management for Treatment Attendance: Cibler l’engagement en matière de traitement des dépendances : essai d’efficacité à répartition aléatoire mené au Canada portant sur la gestion des contingences mise en œuvre à distance pour la participation au traitement Supplemental material, sj-docx-1-cpa-10.1177_07067437261477550 for Targeting Addiction Treatment Engagement: A Canadian Randomized Effectiveness Trial of Remotely Administered Contingency Management for Treatment Attendance: Cibler l’engagement en matière de traitement des dépendances : essai d’efficacité à répartition aléatoire mené au Canada portant sur la gestion des contingences mise en œuvre à distance pour la participation au traitement by David C. Hodgins, Jane Yi, Angela Wallace, Laurence Macrae Lynch, Ashley Ethier, Luija Long and Megan E. Cowie in The Canadian Journal of Psychiatry

sj-docx-2-cpa-10.1177_07067437261477550 - Supplemental material for Targeting Addiction Treatment Engagement: A Canadian Randomized Effectiveness Trial of Remotely Administered Contingency Management for Treatment Attendance: Cibler l’engagement en matière de traitement des dépendances : essai d’efficacité à répartition aléatoire mené au Canada portant sur la gestion des contingences mise en œuvre à distance pour la participation au traitement Supplemental material, sj-docx-2-cpa-10.1177_07067437261477550 for Targeting Addiction Treatment Engagement: A Canadian Randomized Effectiveness Trial of Remotely Administered Contingency Management for Treatment Attendance: Cibler l’engagement en matière de traitement des dépendances : essai d’efficacité à répartition aléatoire mené au Canada portant sur la gestion des contingences mise en œuvre à distance pour la participation au traitement by David C. Hodgins, Jane Yi, Angela Wallace, Laurence Macrae Lynch, Ashley Ethier, Luija Long and Megan E. Cowie in The Canadian Journal of Psychiatry
