# Supplementary material for: Targeting Addiction Treatment Engagement: A Canadian Randomized Effectiveness Trial of Remotely Administered Contingency Management for Treatment Attendance: Cibler l’engagement en matière de traitement des dépendances : essai d’efficacité à répartition aléatoire mené au Canada portant sur la gestion des contingences mise en œuvre à distance pour la participation au traitement
Source: Can J Psychiatry. 2026 Aug 27:07067437261477550. Online ahead of print. doi: 10.1177/07067437261477550 (PMC13522263; doi:10.1177/07067437261477550)
Supplement: Supplementary file 1 [file sj-docx-1-cpa-10.1177_07067437261477550.docx]

**Supplementary Files**

CM Schedules Examples (Tables S1-S5)

**Table S1**

*Participant Scheduled for One TAU Session on One Day of the Week*

| Week of Intervention | M* | T | W | TH | F | Bonus for Attending All Sessions | Total Weekly Draws |
| --- | --- | --- | --- | --- | --- | --- | --- |
|  | Number of Draws | | | | | | |
| Week 1 | 1 |  |  |  |  | **0** | 1 |
| Week 2 | 2 |  |  |  |  | **0** | 2 |
| Week 3 | 3 |  |  |  |  | **0** | 3 |
| Week 4 | 4 |  |  |  |  | **0** | 4 |
| Week 5 | 5 |  |  |  |  | **0** | 5 |
| Week 6 | 6 |  |  |  |  | **0** | 6 |
| Week 7 | 7 |  |  |  |  | **0** | 7 |
| Week 8 | 8 |  |  |  |  | **0** | 8 |
| Week 9 | 9 |  |  |  |  | **0** | 9 |
| Week 10 | 10 |  |  |  |  | **0** | 10 |
| Week 11 | 11 |  |  |  |  | **0** | 11 |
| Week 12 | 12 |  |  |  |  | **0** | 12 |

*Note.* *Indicates a prize draw day. TAU=treatment-as-usual. Bolded values indicate a bonus draw.

**Table S2**

| Week of Intervention | M* | T | W | TH | F | Bonus for Attending All Sessions | Total Weekly Draws |
| --- | --- | --- | --- | --- | --- | --- | --- |
|  | Number of Draws | | | | | | |
| Week 1 | 1+2 |  |  |  |  | **0** | 3 |
| Week 2 | 3+4 |  |  |  |  | **0** | 7 |
| Week 3 | 5+6 |  |  |  |  | **0** | 11 |
| Week 4 | 7+7 |  |  |  |  | **0** | 14 |
| Week 5 | 7+7 |  |  |  |  | **0** | 14 |
| Week 6 | 7+7 |  |  |  |  | **0** | 14 |
| Week 7 | 7+7 |  |  |  |  | **0** | 14 |
| Week 8 | 7+7 |  |  |  |  | **0** | 14 |
| Week 9 | 7+7 |  |  |  |  | **0** | 14 |
| Week 10 | 7+7 |  |  |  |  | **0** | 14 |
| Week 11 | 7+7 |  |  |  |  | **0** | 14 |
| Week 12 | 7+7 |  |  |  |  | **0** | 14 |

*Participant Scheduled for Two TAU Sessions on One Day of the Week*

*Note.* *Indicates a prize draw day. TAU=treatment-as-usual. Bolded values indicate a bonus draw.

**Table S3**

*Participant Scheduled for Three or More TAU Sessions on One Day of the Week*

| Week of Intervention | M* | T | W | TH | F | Bonus for Attending All Sessions | Total Weekly Draws |
| --- | --- | --- | --- | --- | --- | --- | --- |
|  | Number of Draws | | | | | | |
| Week 1 | 1+2**+1** |  |  |  |  | **1** | 4 |
| Week 2 | 3+4**+1** |  |  |  |  | **1** | 8 |
| Week 3 | 5+6**+1** |  |  |  |  | **1** | 12 |
| Week 4 | 7+7**+1** |  |  |  |  | **1** | 15 |
| Week 5 | 7+7**+1** |  |  |  |  | **1** | 15 |
| Week 6 | 7+7**+1** |  |  |  |  | **1** | 15 |
| Week 7 | 7+7**+1** |  |  |  |  | **1** | 15 |
| Week 8 | 7+7**+1** |  |  |  |  | **1** | 15 |
| Week 9 | 7+7**+1** |  |  |  |  | **1** | 15 |
| Week 10 | 7+7**+1** |  |  |  |  | **1** | 15 |
| Week 11 | 7+7**+1** |  |  |  |  | **1** | 15 |
| Week 12 | 7+7**+1** |  |  |  |  | **1** | 15 |

*Note.* *Indicates a prize draw day. TAU=treatment-as-usual. Bolded values indicate a bonus draw.

**Table S4**

*Participant Scheduled for Two TAU Sessions on Two Days of the Week.*

*Note.* *Indicates a prize draw day. TAU=treatment-as-usual. Bolded values indicate a bonus draw.

| Week of Intervention | M* | T | W | TH* | F | Bonus for Attending All Sessions | Total Weekly Draws |
| --- | --- | --- | --- | --- | --- | --- | --- |
|  | Number of Draws | | | | | | |
| Week 1 | 1 |  |  | 2 |  | **0** | 3 |
| Week 2 | 3 |  |  | 4 |  | **0** | 7 |
| Week 3 | 5 |  |  | 6 |  | **0** | 11 |
| Week 4 | 7 |  |  | 7 |  | **0** | 14 |
| Week 5 | 7 |  |  | 7 |  | **0** | 14 |
| Week 6 | 7 |  |  | 7 |  | **0** | 14 |
| Week 7 | 7 |  |  | 7 |  | **0** | 14 |
| Week 8 | 7 |  |  | 7 |  | **0** | 14 |
| Week 9 | 7 |  |  | 7 |  | **0** | 14 |
| Week 10 | 7 |  |  | 7 |  | **0** | 14 |
| Week 11 | 7 |  |  | 7 |  | **0** | 14 |
| Week 12 | 7 |  |  | 7 |  | **0** | 14 |

**Table S5**

| Week of Intervention | M* | T | W | TH* | F | Bonus for Attending All Sessions | Total Weekly Draws |
| --- | --- | --- | --- | --- | --- | --- | --- |
|  | Number of Draws | | | | | | |
| Week 1 | 1 |  | Attended | 2**+1** |  | **1** | 4 |
| Week 2 | 3 |  | Attended | 4**+1** |  | **1** | 8 |
| Week 3 | 5 |  | Attended | 6**+1** |  | **1** | 12 |
| Week 4 | 7 |  | Attended | 7**+1** |  | **1** | 15 |
| Week 5 | 7 |  | Attended | 7**+1** |  | **1** | 15 |
| Week 6 | 7 |  | Attended | 7**+1** |  | **1** | 15 |
| Week 7 | 7 |  | Attended | 7**+1** |  | **1** | 15 |
| Week 8 | 7 |  | Attended | 7**+1** |  | **1** | 15 |
| Week 9 | 7 |  | Attended | 7**+1** |  | **1** | 15 |
| Week 10 | 7 |  | Attended | 7**+1** |  | **1** | 15 |
| Week 11 | 7 |  | Attended | 7**+1** |  | **1** | 15 |
| Week 12 | 7 |  | Attended | 7**+1** |  | **1** | 15 |

*Participant Scheduled for Three or More TAU Sessions on Three or More Days of the Week*

*Note.* *Indicates a prize draw day. TAU=treatment-as-usual. Bolded values indicate a bonus draw.

**Table S6**

*Measures Obtained Over the Three-Month Study Period*

|  | | | **Screening** | | **Intervention Period** | | | | | | | | | | | | **Follow-up** | |
| --- | --- | --- | --- | --- | --- | --- | --- | --- | --- | --- | --- | --- | --- | --- | --- | --- | --- | --- |
| **Month** | | |  | | **1** | | | | **2** | | | | **3** | | | | **3,6,12 Months** | |
| **Week** | | |  | | **1** | **2** | **3** | **4** | **5** | **6** | **7** | **8** | **9** | **10** | **11** | **12** |  |  |
| **Client Measures** | | |  | |  |  |  |  |  |  |  |  |  |  |  |  |  |  |
| Informed consent | | | X | |  |  |  |  |  |  |  |  |  |  |  |  |  |  |
| Demographics | | | X | |  |  |  |  |  |  |  |  |  |  |  |  |  |  |
| Gambling problems | | | X | |  |  |  |  |  |  |  |  |  |  |  |  |  |  |
|  | *NODS* | |  |  |  |  |  |  |  |  |  |  |  |  |  |  |  |  |
| Substance use | | |  | |  |  |  |  |  |  |  |  |  |  |  |  |  |  |
|  | *TLFB* | | X | | X | X | X | X | X | X | X | X | X | X | X | X | X |  |
| Quality of life | | | X | |  | X |  | X |  | X |  | X |  | X |  | X |  |  |
|  | *WHOQOL-BREF* | |  |  |  |  |  |  |  |  |  |  |  |  |  |  | X |  |
| **Staff Measures** | | |  | |  |  |  |  |  |  |  |  |  |  |  |  |  |  |
| Tracking Attendance and CM | | |  | |  |  |  |  |  |  |  |  |  |  |  |  |  |  |
|  | Tracking TAU & CM delivery | |  | | X | X | X | X | X | X | X | X | X | X | X | X |  |  |
|  |  | Retention |  | | X | X | X | X | X | X | X | X | X | X | X | X |  |  |
|  |  | Attendance |  | | X | X | X | X | X | X | X | X | X | X | X | X |  |  |
|  | Incentive inventory log | |  | | X | X | X | X | X | X | X | X | X | X | X | X |  |  |
|  | Session, treatment, and incentive reminder form | | |  | X | X | X | X | X | X | X | X | X | X | X | X |  |  |

*Note.* NODS=National Opinion Research Center DSM Screen for Gambling Problems; TLFB=Timeline Followback; WHOQOL-BREF=World Health Organization Quality of Life-BREF; TAU=treatment-as-usual; CM=contingency management.

**Table S7**

*Baseline Demographic Characteristics of Participants by Recruitment Site*

| Baseline characteristics | | | | Calgary  (n=49) | | | | | | | | Edmonton  (n=39) | | | | | | Difference at baseline | | | |  |
| --- | --- | --- | --- | --- | --- | --- | --- | --- | --- | --- | --- | --- | --- | --- | --- | --- | --- | --- | --- | --- | --- | --- |
|  |  |  |  | *n* | | % | | *M (SD)* | | | | *n* | % | | | *M (SD)* | | Test, *df* | | *p* | |  |
| Age^a^ | | | | 49 | |  | | 36.47 (10.42) | | | | 39 |  | | | 37.49 (11.84) | | 943.50 | | .920 | |  |
| Sex^b^ | | | |  | |  | |  | | | |  |  | | |  | |  | |  | |  |
|  | | Female | | 30 | | 61.22 | |  | | | | 18 | 46.15 | | |  | | 1.99, 1 | | .158 | |  |
|  | | Male | | 19 | | 38.78 | |  | | | | 21 | 53.85 | | |  | |  |  |  |  |  |
| Gender^c^ | | | |  | |  | |  | | | |  |  | | |  | |  | |  | |  |
|  | | Female | | 29 | | 59.18 | |  | | | | 17 | 43.59 | | |  | | 2.35 | | .310 | |  |
|  | | Male | | 19 | | 38.78 | |  | | | | 21 | 53.85 | | |  | |  |  |  |  |  |
|  | | Other | | 1 | | 2.04 | |  | | | | 1 | 2.56 | | |  | |  |  |  |  |  |
| Marital status^c^ | | | |  | |  | |  | | | |  |  | | |  | |  | |  | |  |
|  | | Single | | 27 | | 55.10 | |  | | | | 24 | 61.54 | | |  | | 2.84 | | .412 | |  |
|  | | Married | | 1 | | 2.04 | |  | | | | 3 | 7.69 | | |  | |  |  |  |  |  |
|  | | Common law | | 15 | | 30.61 | |  | | | | 8 | 20.51 | | |  | |  |  |  |  |  |
|  | | Separated/divorced/  widowed | | 6 | | 12.24 | |  | | | | 3 | 7.69 | | |  | |  |  |  |  |  |
|  | | Missing | | 0 | | 0.00 | |  | | | | 1 | 2.56 | | |  | |  |  |  |  |  |
| Ethnicity^c^ | | | |  | |  | |  | | | |  |  | | |  | |  | |  | |  |
|  | | Indigenous | | 9 | | 18.37 | |  | | | | 10 | 25.64 | | |  | | 3.94 | | .422 | |  |
|  | | Black | | 1 | | 2.04 | |  | | | | 1 | 2.56 | | |  | |  |  |  |  |  |
|  | | White | | 33 | | 67.35 | |  | | | | 26 | 66.67 | | |  | |  |  |  |  |  |
|  | | Asian | | 4 | | 8.16 | |  | | | | 0 | 0.00 | | |  | |  |  |  |  |  |
|  | | Latin American | | 2 | | 4.08 | |  | | | | 1 | 2.56 | | |  | |  |  |  |  |  |
|  | | Missing | | 0 | | 0.00 | |  | | | | 1 | 2.56 | | |  | |  |  |  |  |  |
| Highest level of education^c^ | | | |  | |  | |  | | | |  |  | | |  | |  | |  | |  |
|  | | Highschool or lower | | 19 | | 38.78 | |  | | | | 19 | 48.72 | | |  | | 1.77 | | .650 | |  |
|  | | Trades or College | | 16 | | 32.65 | |  | | | | 12 | 30.77 | | |  | |  |  |  |  |  |
|  | | Bachelor’s or diploma below | | 11 | | 22.45 | |  | | | | 5 | 12.82 | | |  | |  |  |  |  |  |
|  | | Master’s or Doctorate | | 3 | | 6.12 | |  | | | | 3 | 7.69 | | |  | |  |  |  |  |  |
| Employment status^c^ | | | |  | |  | |  | | | |  |  | | |  | |  | |  | |  |
|  | | Employed | | 22 | | 44.90 | |  | | | | 14 | 35.90 | | |  | | 2.77 | | .450 | |  |
|  | | Unemployed | | 18 | | 36.73 | |  | | | | 13 | 33.33 | | |  | |  |  |  |  |  |
|  | | Student | | 4 | | 8.16 | |  | | | | 3 | 7.69 | | |  | |  |  |  |  |  |
|  | | Retired/Disabled/  Leave | | 5 | | 10.20 | |  | | | | 9 | 23.08 | | |  | |  |  |  |  |  |
| Household income before tax^c^ | | | |  | |  | |  | | | |  |  | | |  | |  | |  | |  |
|  | | $0-$9,999 | | 3 | | 6.12 | |  | | | | 3 | 7.69 | | |  | | 1.03 | | .999 | |  |
|  | | $10,000-$19,999 | | 5 | | 10.20 | |  | | | | 4 | 10.26 | | |  | |  |  |  |  |  |
|  | | $20,000-$39,999 | | 12 | | 24.49 | |  | | | | 10 | 25.64 | | |  | |  |  |  |  |  |
|  | | $40,000-$59,999 | | 6 | | 12.24 | |  | | | | 5 | 12.82 | | |  | |  |  |  |  |  |
|  | | $60,000-$79,000 | | 5 | | 10.20 | |  | | | | 4 | 10.26 | | |  | |  |  |  |  |  |
|  | | $80,000-$99,000 | | 3 | | 6.12 | |  | | | | 1 | 2.56 | | |  | |  |  |  |  |  |
|  | | $100,000+ | | 7 | | 14.29 | |  | | | | 6 | 15.38 | | |  | |  |  |  |  |  |
|  | | Unsure | | 8 | | 16.33 | |  | | | | 6 | 15.38 | | |  | |  |  |  |  |  |
| Substance use | | | |  | |  | |  | | | |  |  | | |  | |  | |  | |  |
| Alcohol use disorder | | | 61 | | 69.13 | |  | | 26 | 66.70 |  | | | 35 | 71.40 | |  | | 0.23 | | .630 | |
| Cannabis use disorder | | | 13 | | 14.80 | |  | | 7 | 17.90 |  | | | 6 | 12.20 | |  | | 0.56 | | .454 | |
|  | Opioid use disorder | | 8 | | 9.10 | |  | | 4 | 10.30 |  | | | 4 | 10.30 | |  | | 0.12 | | .734 | |
|  | Stimulant use disorder | | 36 | | 40.90 | |  | | 15 | 38.50 |  | | | 21 | 42.90 | |  | | .17 | | .677 | |
|  | Other SUD | | 4 | | 4.54 | |  | | 2 | 4.70 |  | | | 2 | 4.40 | |  | | 0.01 | | .929 | |
|  | | Longest consecutive days abstinent^d^ | | 49 | |  | | 5.49 (0.12) | | | | 39 |  | | | 4.79 (0.13) | | 0.37, 1 | | .541 | |  |
|  | | Longest consecutive days of substance use^d^ | | 49 | |  | | 10.51 (0.22) | | | | 39 |  | | | 18.38 (0.48) | | 6.63, 1 | | .010 | |  |
|  | | Mean standard drinks^d^ | | 49 | |  | | 9.61 (0.21) | | | | 39 |  | | | 9.79 (0.26) | | 0.01, 1 | | .933 | |  |
|  | | Total number of standard drinks^d^ | | 49 | |  | | 127.19 (2.60) | | | | 39 |  | | | 116.09 (2.98) | | 0.18, 1 | | .671 | |  |
|  | | Largest number of standard drinks^d^ | | 49 | |  | | 13.00 (0.28) | | | | 39 |  | | | 13.23 (0.35) | | 0.01, 1 | | .937 | |  |
|  | | Proportion of days abstinent^a^ | | 49 | |  | | 0.41 (0.30) | | | | 39 |  | | | 0.29 (0.33) | | 689.50 | | .025 | |  |
| WHOQOL-BREF | | | |  | |  | |  | | | |  |  | | |  | |  | |  | |  |
|  | | Environment^a^ | | 49 | |  | | 65.25 (17.27) | | | | 39 |  | | | 69.47 (14.38) | | 851.50 | | .381 | |  |
|  | | Social^a^ | | 49 | |  | | 54.59 (22.44) | | | | 39 |  | | | 52.35 (22.37) | | 926.00 | | .803 | |  |
|  | | Physical health^e^ | | 49 | |  | | 61.30 (18.49) | | | | 39 |  | | | 56.96 (18.75) | | 1.09, 86 | | .280 | |  |
|  | | Psychological^e^ | | 49 | |  | | 49.92 (18.91) | | | | 39 |  | | | 44.02 (21.11) | | 1.38, 86 | | .171 | |  |

*Note.*^a^=Mann-Whitney U Test; ^b^=Chi Square Test; ^c^=Fisher’s Exact Test; ^d^=Negative Binomial Regression; ^e^=Independent Samples t-test.

**Table S8**

*Demographic Characteristics of Participants by Completers and Non-Completers*

| Baseline characteristics | | | | | Completers  (n=46) | | | | | | Non-Completers  (n=42) | | | | | | Difference at baseline | | | | | |  |
| --- | --- | --- | --- | --- | --- | --- | --- | --- | --- | --- | --- | --- | --- | --- | --- | --- | --- | --- | --- | --- | --- | --- | --- |
|  |  |  |  |  | *n* | % | | *M (SD)* | | | *n* | | % | | *M (SD)* | | | | Test, *df* | | *p* | | |
| Group^a^ | | | | |  |  | |  | | |  | |  | |  | | | |  | |  | | |
|  | | CM | | | 26 | 56.52 | |  | | | 17 | | 40.48 | |  | | | | 2.26, 1 | | .133 | | |
|  | | TAU | | | 20 | 43.48 | |  | | | 25 | | 59.52 | |  | | | |  |  |  |  |  |
| Site^a^ | | | | |  |  | |  | | |  | |  | |  | | | |  | |  | | |
|  | | Calgary | | | 22 | 47.83 | |  | | | 27 | | 64.29 | |  | | | | 2.41, 1 | | .121 | | |
|  | | Edmonton | | | 24 | 52.17 | |  | | | 15 | | 35.71 | |  | | | |  |  |  |  |  |
| Age^b^ | | | | | 46 |  | | 39.39 (11.59) | | | 42 | |  | | 34.21 (9.78) | | | | 695.50 | | .024 | | |
| Sex^a^ | | | | |  |  | |  | | |  | |  | |  | | | |  | |  | | |
|  | | Female | | | 24 | 52.17 | |  | | | 24 | | 57.14 | |  | | | | 0.22, 1 | | .640 | | |
|  | | Male | | | 22 | 47.83 | |  | | | 18 | | 42.86 | |  | | | |  |  |  |  |  |
| Gender^c^ | | | | |  |  | |  | | |  | |  | |  | | | |  | |  | | |
|  | | Female | | | 22 | 47.83 | |  | | | 24 | | 57.14 | |  | | | | 1.95 | | .431 | | |
|  | | Male | | | 22 | 47.83 | |  | | | 18 | | 42.86 | |  | | | |  |  |  |  |  |
|  | | Other | | | 2 | 4.35 | |  | | | 0 | | 0.00 | |  | | | |  |  |  |  |  |
| Marital status^c^ | | | | |  |  | |  | | |  | |  | |  | | | |  | |  | | |
|  | | Single | | | 27 | 58.70 | |  | | | 24 | | 57.14 | |  | | | | 8.36 | | .031 | | |
|  | | Married | | | 4 | 8.70 | |  | | | 0 | | 0.00 | |  | | | |  |  |  |  |  |
|  | | Common law | | | 8 | 17.39 | |  | | | 15 | | 35.71 | |  | | | |  |  |  |  |  |
|  | | Separated/divorced/widowed | | | 7 | 15.22 | |  | | | 2 | | 4.76 | |  | | | |  |  |  |  |  |
|  | | Missing | | | 0 | 0.00 | |  | | | 1 | | 2.38 | |  | | | |  |  |  |  |  |
| Ethnicity^c^ | | | | |  |  | |  | | |  | |  | |  | | | |  | |  | | |
|  | | Indigenous | | | 6 | 13.04 | |  | | | 13 | | 30.95 | |  | | | | 7.43 | | .070 | | |
|  | | Black | | | 0 | 0.00 | |  | | | 2 | | 4.76 | |  | | | |  |  |  |  |  |
|  | | White | | | 35 | 76.09 | |  | | | 24 | | 57.14 | |  | | | |  |  |  |  |  |
|  | | Asian | | | 3 | 6.52 | |  | | | 1 | | 2.38 | |  | | | |  |  |  |  |  |
|  | | Latin American | | | 1 | 2.17 | |  | | | 2 | | 4.76 | |  | | | |  |  |  |  |  |
|  | | Missing | | | 1 | 2.17 | |  | | | 0 | | 0.00 | |  | | | |  |  |  |  |  |
| Highest level of education^c^ | | | | |  |  | |  | | |  | |  | |  | | | |  | |  | | |
|  | | Highschool or lower | | | 16 | 34.78 | |  | | | 22 | | 52.38 | |  | | | | 2.95 | | .427 | | |
|  | | Trades or College | | | 17 | 36.96 | |  | | | 11 | | 26.19 | |  | | | |  |  |  |  |  |
|  | | Bachelor’s or diploma below | | | 9 | 19.57 | |  | | | 7 | | 16.67 | |  | | | |  |  |  |  |  |
|  | | Master’s or Doctorate | | | 4 | 8.70 | |  | | | 2 | | 4.76 | |  | | | |  |  |  |  |  |
| Employment status^c^ | | | | |  |  | |  | | |  | |  | |  | | | |  | |  | | |
|  | | Employed | | | 16 | 34.78 | |  | | | 20 | | 28.57 | |  | | | | 2.06 | | .581 | | |
|  | | Unemployed | | | 19 | 41.30 | |  | | | 12 | | 47.62 | |  | | | |  |  |  |  |  |
|  | | Student | | | 4 | 8.70 | |  | | | 3 | | 7.14 | |  | | | |  |  |  |  |  |
|  | | Retired/Disabled/Leave | | | 7 | 15.22 | |  | | | 7 | | 16.67 | |  | | | |  |  |  |  |  |
| Household income before tax^c^ | | | | |  |  | |  | | |  | |  | |  | | | |  | |  | | |
|  | | $0-$9,999 | | | 5 | 10.87 | |  | | | 1 | | 2.38 | |  | | | | 5.14 | | .667 | | |
|  | | $10,000-$19,999 | | | 4 | 8.70 | |  | | | 5 | | 11.90 | |  | | | |  |  |  |  |  |
|  | | $20,000-$39,999 | | | 9 | 19.57 | |  | | | 13 | | 30.95 | |  | | | |  |  |  |  |  |
|  | | $40,000-$59,999 | | | 5 | 10.87 | |  | | | 6 | | 14.29 | |  | | | |  |  |  |  |  |
|  | | $60,000-$79,000 | | | 5 | 10.87 | |  | | | 4 | | 9.52 | |  | | | |  |  |  |  |  |
|  | | $80,000-$99,000 | | | 3 | 6.52 | |  | | | 1 | | 2.38 | |  | | | |  |  |  |  |  |
|  | | $100,000+ | | | 8 | 17.39 | |  | | | 5 | | 11.90 | |  | | | |  |  |  |  |  |
|  | | Unsure | | | 7 | 15.22 | |  | | | 7 | | 16.67 | |  | | | |  |  |  |  |  |
| Substance use | | | | |  |  | |  | | |  | |  | |  | | | |  | |  | | |
| Alcohol use disorder | | |  |  |  | | 32 | | 69.60 |  | | 29 | | 69.00 | |  | | 0.003 | | .958 | |  |  |
| Cannabis use disorder | | |  |  |  | | 8 | | 13.00 |  | | 7 | | 16.70 | |  | | 0.23 | | ..632 | |  |  |
|  | Opioid use disorder | |  |  |  | | 3 | | 6.50 |  | | 5 | | 11.90 | |  | | 0.77 | | .380 | |  |  |
|  | Stimulant use disorder | |  |  |  | | 17 | | 37.00 |  | | 19 | | 45.20 | |  | | 0.62 | | .430 | |  |  |
|  | Other SUD | |  |  |  | | 2 | | 4.76 |  | | 2 | | 4.40 | |  | | 0.01 | | .929 | |  |  |
|  | | | | |  |  | |  | | |  | |  | |  | | | |  | |  | | |
|  | | Longest consecutive days abstinent^d^ | | | 46 |  | | 6.05 (0.14) | | | 42 | |  | | 3.97 (0.10) | | | | 3.65, 1 | | .056 | | |
|  | | Longest consecutive days of substance use^d^ | | | 46 |  | | 13.52 (0.30) | | | 42 | |  | | 14.52 (0.35) | | | | 0.11, 1 | | .741 | | |
|  | | Mean standard drinks^d^ | | | 46 |  | | 9.28 (0.21) | | | 42 | |  | | 10.14 (0.25) | | | | 0.16, 1 | | .693 | | |
|  | | Total number of standard drinks^d^ | | | 46 |  | | 108.49 (2.36) | | | 42 | |  | | 137.37 (3.27) | | | | 1.22, 1 | | .269 | | |
|  | | Largest number of standard drinks^d^ | | | 46 |  | | 13.13 (0.30) | | | 42 | |  | | 13.07 (0.32) | | | | 0.00, 1 | | .984 | | |
|  | | Proportion of days abstinent^b^ | | | 46 |  | | 0.40 (0.32) | | | 42 | |  | | 0.31 (0.33) | | | | 819.00 | | .217 | | |
| WHOQOL-BREF | | | | |  |  | |  | | |  | |  | |  | | | |  | |  | | |
|  | | Environment^b^ | | | 46 |  | | 70.11 (14.35) | | | 42 | |  | | 63.85 (17.42) | | | | 748.50 | | .068 | | |
|  | | Social^b^ | | | 46 |  | | 57.04 (21.42) | | | 42 | |  | | 50.35 (21.79) | | | | 771.50 | | .101 | | |
|  | | Physical health^e^ | | | 46 |  | | 61.34 (19.33) | | | 42 | |  | | 57.23 (17.80) | | | | -1.03, 86 | | .304 | | |
|  | | Psychological^e^ | | | 46 |  | | 48.64 (19.80) | | | 42 | |  | | 45.83 (20.39) | | | | -0.66, 86 | | .514 | | |

*Note.*^a^=Chi Square Test; ^b^=Mann-Whitney U Test; ^c^=Fishers Exact Test; ^d^=Negative Binomial Regression; ^e^=Independent Samples t-test. CM=Contingency Management, TAU=Treatment-as-usual.

**Table S9**

*GEE for WHOQOL-BREF Quality of Life Outcomes Across Time*

| Effect | | IRR | Estimate | *SE* | *df* | Wald Chi-Square | *p* | 95% CI | |
| --- | --- | --- | --- | --- | --- | --- | --- | --- | --- |
|  |  |  |  |  |  |  |  | LL | UL |
| Physical health | |  |  |  |  |  |  |  |  |
|  | Intercept | 61.48 | 4.12 | 0.07 | 1 | 3379.62 | <.001 | 3.98 | 4.26 |
|  | Group: CM | 1.06 | 0.06 | 0.07 | 1 | 0.83 | .362 | -0.07 | 0.19 |
|  | Site: Edmonton | 0.96 | -0.04 | 0.06 | 1 | 0.45 | .502 | -0.15 | 0.07 |
|  | Sex: Female | 0.91 | -0.09 | 0.06 | 1 | 2.43 | .119 | -0.20 | 0.02 |
|  | Time: Week 2 | 1.02 | 0.02 | 0.04 | 1 | 0.16 | .685 | -0.06 | 0.09 |
|  | Time: Week 4 | 1.12 | 0.11 | 0.06 | 1 | 3.77 | .052 | 0.00 | 0.22 |
|  | Time: Week 6 | 1.07 | 0.06 | 0.05 | 1 | 1.88 | .171 | -0.03 | 0.16 |
|  | Time: Week 8 | 1.06 | 0.06 | 0.05 | 1 | 1.43 | .232 | -0.04 | 0.16 |
|  | Time: Week 10 | 1.03 | 0.03 | 0.04 | 1 | 0.36 | .547 | -0.06 | 0.11 |
|  | Time: Week 12 | 1.08 | 0.08 | 0.06 | 1 | 1.61 | .205 | -0.04 | 0.20 |
|  | Time: Period 1 | 1.06 | 0.06 | 0.06 | 1 | 0.94 | .333 | -0.06 | 0.19 |
|  | Time: Period 2 | 1.13 | 0.12 | 0.07 | 1 | 3.11 | .078 | -0.01 | 0.25 |
|  | Time: Period 3 | 1.14 | 0.13 | 0.05 | 1 | 7.28 | .007 | 0.04 | 0.22 |
|  | Group*Time: Week 2 | 1.02 | 0.02 | 0.05 | 1 | 0.27 | .602 | -0.07 | 0.12 |
|  | Group*Time: Week 4 | 0.94 | -0.06 | 0.07 | 1 | 0.77 | .381 | -0.19 | 0.07 |
|  | Group*Time: Week 6 | 1.03 | 0.03 | 0.06 | 1 | 0.26 | .610 | -0.08 | 0.14 |
|  | Group*Time: Week 8 | 1.03 | 0.03 | 0.06 | 1 | 0.21 | .643 | -0.09 | 0.14 |
|  | Group*Time: Week 10 | 1.10 | 0.09 | 0.06 | 1 | 2.11 | .147 | -0.03 | 0.22 |
|  | Group*Time: Week 12 | 1.06 | 0.06 | 0.07 | 1 | 0.68 | .408 | -0.08 | 0.20 |
|  | Group*Time: Period 1 | 1.09 | 0.09 | 0.09 | 1 | 1.06 | .304 | -0.08 | 0.26 |
|  | Group*Time: Period 2 | 1.01 | 0.01 | 0.08 | 1 | 0.03 | .852 | -0.14 | 0.16 |
|  | Group*Time: Period 3 | 1.02 | 0.02 | 0.08 | 1 | 0.08 | .772 | -0.13 | 0.17 |
| Psychological | |  |  |  |  |  |  |  |  |
|  | Intercept | 48.51 | 3.88 | 0.08 | 1 | 2090.16 | <.001 | 3.72 | 4.05 |
|  | Group: CM | 1.17 | 0.16 | 0.09 | 1 | 2.91 | .088 | -0.02 | 0.33 |
|  | Site: Edmonton | 0.94 | -0.07 | 0.08 | 1 | 0.71 | .400 | -0.22 | 0.09 |
|  | Sex: Female | 0.87 | -0.14 | 0.08 | 1 | 3.33 | .068 | -0.29 | 0.01 |
|  | Time: Week 2 | 1.16 | 0.15 | 0.06 | 1 | 6.52 | .011 | 0.03 | 0.26 |
|  | Time: Week 4 | 1.23 | 0.21 | 0.06 | 1 | 12.81 | .000 | 0.09 | 0.32 |
|  | Time: Week 6 | 1.29 | 0.25 | 0.06 | 1 | 17.34 | .000 | 0.13 | 0.37 |
|  | Time: Week 8 | 1.25 | 0.22 | 0.07 | 1 | 9.08 | .003 | 0.08 | 0.37 |
|  | Time: Week 10 | 1.14 | 0.13 | 0.10 | 1 | 1.85 | .174 | -0.06 | 0.32 |
|  | Time: Week 12 | 1.27 | 0.24 | 0.08 | 1 | 7.97 | .005 | 0.07 | 0.40 |
|  | Time: Period 1 | 1.18 | 0.16 | 0.10 | 1 | 2.73 | .099 | -0.03 | 0.35 |
|  | Time: Period 2 | 1.20 | 0.18 | 0.10 | 1 | 3.56 | .059 | -0.01 | 0.37 |
|  | Time: Period 3 | 1.22 | 0.20 | 0.12 | 1 | 2.81 | .094 | -0.03 | 0.42 |
|  | Group*Time: Week 2 | 0.92 | -0.08 | 0.07 | 1 | 1.36 | .244 | -0.22 | 0.06 |
|  | Group*Time: Week 4 | 0.90 | -0.10 | 0.07 | 1 | 2.04 | .153 | -0.24 | 0.04 |
|  | Group*Time: Week 6 | 0.89 | -0.11 | 0.08 | 1 | 2.11 | .147 | -0.27 | 0.04 |
|  | Group*Time: Week 8 | 0.88 | -0.13 | 0.09 | 1 | 2.12 | .146 | -0.31 | 0.05 |
|  | Group*Time: Week 10 | 0.98 | -0.02 | 0.11 | 1 | 0.03 | .853 | -0.23 | 0.19 |
|  | Group*Time: Week 12 | 0.89 | -0.11 | 0.10 | 1 | 1.38 | .241 | -0.31 | 0.08 |
|  | Group*Time: Period 1 | 1.03 | 0.03 | 0.11 | 1 | 0.09 | .762 | -0.19 | 0.25 |
|  | Group*Time: Period 2 | 1.00 | <0.01 | 0.11 | 1 | <0.01 | .967 | -0.21 | 0.22 |
|  | Group*Time: Period 3 | 0.94 | -0.06 | 0.14 | 1 | 0.17 | .678 | -0.34 | 0.22 |
| Social | |  |  |  |  |  |  |  |  |
|  | Intercept | 53.73 | 3.98 | 0.07 | 1 | 2901.61 | <.001 | 3.84 | 4.13 |
|  | Group: CM | 0.89 | -0.12 | 0.09 | 1 | 1.97 | .160 | -0.29 | 0.05 |
|  | Site: Edmonton | 1.07 | 0.06 | 0.07 | 1 | 0.91 | .340 | -0.07 | 0.20 |
|  | Sex: Female | 1.06 | 0.06 | 0.07 | 1 | 0.70 | .403 | -0.08 | 0.19 |
|  | Time: Week 2 | 1.09 | 0.09 | 0.07 | 1 | 1.71 | .192 | -0.04 | 0.21 |
|  | Time: Week 4 | 1.07 | 0.07 | 0.07 | 1 | 1.00 | .317 | -0.07 | 0.21 |
|  | Time: Week 6 | 1.10 | 0.09 | 0.07 | 1 | 1.71 | .191 | -0.05 | 0.24 |
|  | Time: Week 8 | 1.14 | 0.13 | 0.08 | 1 | 2.24 | .134 | -0.04 | 0.29 |
|  | Time: Week 10 | 1.03 | 0.03 | 0.08 | 1 | 0.18 | .671 | -0.12 | 0.19 |
|  | Time: Week 12 | 1.08 | 0.08 | 0.09 | 1 | 0.76 | .383 | -0.10 | 0.25 |
|  | Time: Period 1 | 1.03 | 0.03 | 0.09 | 1 | 0.10 | .751 | -0.15 | 0.21 |
|  | Time: Period 2 | 1.00 | 0.00 | 0.12 | 1 | 0.00 | .991 | -0.24 | 0.24 |
|  | Time: Period 3 | 1.00 | 0.00 | 0.09 | 1 | 0.00 | .962 | -0.17 | 0.18 |
|  | Group*Time: Week 2 | 0.97 | -0.03 | 0.09 | 1 | 0.13 | .716 | -0.20 | 0.14 |
|  | Group*Time: Week 4 | 1.03 | 0.03 | 0.09 | 1 | 0.12 | .729 | -0.15 | 0.21 |
|  | Group*Time: Week 6 | 1.11 | 0.10 | 0.10 | 1 | 1.15 | .283 | -0.09 | 0.29 |
|  | Group*Time: Week 8 | 1.00 | <0.01 | 0.10 | 1 | <0.01 | .976 | -0.20 | 0.19 |
|  | Group*Time: Week 10 | 1.10 | 0.10 | 0.11 | 1 | 0.71 | .399 | -0.13 | 0.32 |
|  | Group*Time: Week 12 | 1.06 | 0.06 | 0.11 | 1 | 0.29 | .588 | -0.16 | 0.28 |
|  | Group*Time: Period 1 | 1.14 | 0.13 | 0.12 | 1 | 1.16 | .282 | -0.11 | 0.36 |
|  | Group*Time: Period 2 | 1.15 | 0.14 | 0.14 | 1 | 0.95 | .329 | -0.14 | 0.41 |
|  | Group*Time: Period 3 | 1.10 | 0.10 | 0.12 | 1 | 0.62 | .431 | -0.15 | 0.34 |
| Environment | |  |  |  |  |  |  |  |  |
|  | Intercept | 66.79 | 4.20 | 0.06 | 1 | 5781.74 | <.001 | 4.09 | 4.31 |
|  | Group: CM | 1.01 | 0.01 | 0.05 | 1 | 0.01 | .906 | -0.09 | 0.11 |
|  | Site: Edmonton | 1.06 | 0.05 | 0.05 | 1 | 1.41 | .235 | -0.04 | 0.15 |
|  | Sex: Female | 0.96 | -0.04 | 0.05 | 1 | 0.76 | .383 | -0.14 | 0.05 |
|  | Time: Week 2 | 1.01 | 0.01 | 0.03 | 1 | 0.10 | .750 | -0.06 | 0.08 |
|  | Time: Week 4 | 1.04 | 0.04 | 0.04 | 1 | 0.69 | .408 | -0.05 | 0.12 |
|  | Time: Week 6 | 1.00 | 0.00 | 0.04 | 1 | 0.00 | .951 | -0.09 | 0.09 |
|  | Time: Week 8 | 1.02 | 0.02 | 0.04 | 1 | 0.14 | .709 | -0.07 | 0.10 |
|  | Time: Week 10 | 1.00 | 0.00 | 0.04 | 1 | 0.01 | .934 | -0.08 | 0.07 |
|  | Time: Week 12 | 1.02 | 0.01 | 0.05 | 1 | 0.11 | .743 | -0.07 | 0.10 |
|  | Time: Period 1 | 0.93 | -0.07 | 0.06 | 1 | 1.14 | .285 | -0.19 | 0.06 |
|  | Time: Period 2 | 1.01 | 0.01 | 0.05 | 1 | 0.05 | .817 | -0.09 | 0.11 |
|  | Time: Period 3 | 0.94 | -0.07 | 0.05 | 1 | 1.67 | .197 | -0.17 | 0.03 |
|  | Group*Time: Week 2 | 0.99 | -0.01 | 0.05 | 1 | 0.10 | .751 | -0.10 | 0.07 |
|  | Group*Time: Week 4 | 0.97 | -0.03 | 0.05 | 1 | 0.26 | .610 | -0.13 | 0.08 |
|  | Group*Time: Week 6 | 1.05 | 0.05 | 0.06 | 1 | 0.76 | .383 | -0.06 | 0.16 |
|  | Group*Time: Week 8 | 0.97 | -0.03 | 0.05 | 1 | 0.44 | .505 | -0.14 | 0.07 |
|  | Group*Time: Week 10 | 0.99 | -0.01 | 0.05 | 1 | 0.06 | .809 | -0.11 | 0.09 |
|  | Group*Time: Week 12 | 0.98 | -0.02 | 0.06 | 1 | 0.11 | .737 | -0.13 | 0.09 |
|  | Group*Time: Period 1 | 1.05 | 0.05 | 0.07 | 1 | 0.41 | .520 | -0.09 | 0.18 |
|  | Group*Time: Period 2 | 0.97 | -0.03 | 0.06 | 1 | 0.17 | .682 | -0.15 | 0.10 |
|  | Group*Time: Period 3 | 1.08 | 0.07 | 0.07 | 1 | 0.97 | .325 | -0.07 | 0.22 |

*Note.* Reference categories: TAU (Treatment-as-usual), Calgary, Male, Baseline; IRR=Incidence Rate Ratio; GEE=Generalized Estimating Equation, SE=standard error, *df*=degrees of freedom, CI=95% confidence interval, LL=lower limit, UL=upper limit, WHOQOL-BREF= World Health Organization (WHO) Quality of Life-BREF, CM=contingency management.

**Figure S1**

*Physical Health Quality of Life by Group Across Time*

**Figure S2**

*Psychological Quality of Life by Group Across Time*

**Figure S3**

*Social Quality of Life by Group Across Time*

**Figure S4**

*Environment Quality of Life by Group Across Time*
